# I Can Afford to Relax: Relating Perceived Income Adequacy to Recovery and Health

**DOI:** 10.1007/s41542-024-00200-3

**Published:** 2024-08-14

**Authors:** Kristen Jennings Black, Olivia C. DePhillips, Thomas W. Britt

**Affiliations:** 1 Department of Psychology, The University of Tennessee at Chattanooga, 615 McCallie Ave, Chattanooga, TN 37403 USA; 2 Department of Psychology, Clemson University, Clemson, SC USA

**Keywords:** Recovery experiences, Perceived income adequacy, Financial stress, Relaxation, Occupational stress, Worker health

## Abstract

Employee recovery during non-work hours benefits health in a variety of areas. However, little research has evaluated how recovery may be affected by perceptions of income (in)adequacy. The purpose of the present study was to examine the relationships among workers’ perceived income adequacy (PIA), relaxation remorse, recovery experiences outside of work, and health outcomes. Hypotheses were tested using structural equation modeling with data obtained from a two-wave, time-lagged survey of Amazon’s Mechanical Turk (MTurk) workers (*N* = 683). We found a positive relationship between PIA and recovery, which were both related to better health. PIA was negatively related to relaxation remorse, with relaxation remorse being associated with poor health. There was evidence of indirect relationships via relaxation remorse, where relaxation remorse explained portions of the relationships between PIA and health symptoms. Indirect relationships via recovery experiences were largely unsupported. Our findings expand our understanding of employee recovery as it relates to perceptions of income. Specifically, our studies highlight that one’s perceived income adequacy may be an important contributor to comfort with and/or actual experienced recovery, which can have further health effects.

A large body of literature supports that engaging in relaxation and recovery outside of work has benefits for employee health and well-being (Fritz et al., 2013; Sonnentag & Fritz, 2007; Sonnentag et al., 2017); however, opportunities for recovery may not be as accessible to workers in certain contexts. One’s financial context can affect their thoughts and decisions (Grossman & Mendoza, 2003; Mani et al., 2020; Shah et al., 2018), such as how to allocate time toward work and leisure. Related, economists have noted that along with growing income inequality, there seems to be inequality in consumption and leisure that mirrors the income gap (Attanasio et al., 2015). Low-income workers and those experiencing financial strain have been found to be at risk for experiencing higher rates of physical and mental health concerns across a number of studies and many different populations (e.g., Frank et al., 2014; Prentice et al., 2017; Price et al., 2002), while wealth corresponds with some degree of health and well-being benefits (e.g., Diener & Biswas-Diener, 2002; Hajat et al., 2011). The possible connection between a worker’s perceived financial situation and recovery experiences outside of work have been largely unexplored, but these connections have implications for worker health and well-being.

Therefore, the purpose of the present study was to better understand the potential connections between workers’ income perceptions, recovery experiences, and health. The motivation for this study originated from qualitative responses in the scale development project, for which the present data were obtained (Black & Britt, 2023). In this study, workers responded to items assessing *relaxation remorse*, or feelings of guilt for not engaging in work-related demands during off-work time. Participants were also asked to describe why they responded to the items in the way they did. In a nontrivial number of these responses, individuals expressed that they did not relax because they must continue to work to earn more money for themselves and/or their families, or they were fearful of financial consequences if they chose to relax. While these responses reflected a minority of those given, the sentiments highlighted an important concern—some individuals work excessively and do not engage in recovery out of necessity, not preference. In response to these results, a measure of perceived income adequacy (PIA; Sears, 2008) was included in subsequent data collections of the validation study to secondarily examine how PIA may affect one’s ability to relax and health.

Studies have found that recovery experiences can buffer employees from the effects of economic stressors such as job insecurity (e.g., Kinnunen et al., 2010), but less is known about how income perceptions affect one’s recovery experiences. The quality of recovery is also likely to be impacted by the employee’s mindset toward such activities (e.g., relaxation remorse); however, this topic has received very little research attention. Existing theories around the value and impact of resources (i.e., Conservation of Resources; resource scarcity) suggest that regaining resources through recovery activities may be easier and associated with less guilt for those who have a sufficient supply of financial resources. Along those lines of inquiry, in the present study we examined: (1) whether PIA was associated with health and well-being, (2) whether PIA was related to recovery experiences and relaxation remorse, and (3) whether relaxation remorse and recovery experiences could account for some of the relationships between PIA and health outcomes. We examine these relationships using a two-wave, time-lagged design. In the following sections, we review relevant literature on financial experiences related to well-being and recovery mechanisms, which informed our specific hypotheses.

## Employee’s Perceived Financial Situation and Well-Being

There are several theoretical models that capture the potential health effects that could result from one’s financial stressors and associated resources. For the present study, we frame income, and more specifically the perception of income as adequate, as a resource that could affect workers’ health and well-being, as well as their ability to acquire additional resources through recovery. The Conservation of Resources (COR) theory (Hobfoll, 1989, 2001) is based on the principle that individuals need resources in order to cope with demands. Resources can include objects, personal characteristics, and energies that are valuable. Individuals tend to experience strain when they either do not have enough resources to cope with a demand or feel the threat of loss of valued resources. When resources are threatened, individuals must try to protect existing resources. Protecting and acquiring new resources can be particularly important, because of what Hobfoll (1989, 2001) refers to as loss and gain spirals. That is, losing resources makes individuals susceptible to more resource loss, while gaining resources makes acquiring new resources easier. Money is a critical resource in light of this principle, where losing income can make it difficult to afford necessities while gaining income allows for the acquisition of material (e.g., food, clothing, a home) and nonmaterial (e.g., esteem, a sense of security) resources. Further, the scarcity of income can direct an individual’s thoughts and behaviors toward money/acquiring money (e.g., Shah et al., 2018). Thus, COR theory and scarcity perspectives would suggest that a sufficient income facilitates the acquisition of additional resources, while an inadequate income (or the perception of an inadequate income) would feel threatening and be experienced as strain.

A wealth of research has indeed provided evidence that income can relate to strain, health, and well-being. These relationships have been documented using several operationalizations of economic- or income-related stressors (see Sinclair & Cheung, 2016 for a review on types of measurement). For instance, job insecurity has been related to a number of different health and well-being outcomes (Cheng & Chan, 2008; Shoss, 2017; Sverke et al., 2002), particularly when coinciding with a high degree of job strain (Strazdins et al., 2004). Individuals tend to be vulnerable to health problems when they are unemployed or underemployed (Friedland & Price, 2003; McKee-Ryan et al., 2005). Comparing one’s income to others, such as parents or peers, has also been related to negative effects on well-being (Tibesigwa et al., 2015).

In the present study we sought to replicate these general conclusions that one’s financial situation can affect health. In operationalizing the impact one’s financial situation, we focused on PIA (i.e., perceived ability to afford one’s wants and needs) as a predictor. Studies suggest that PIA is only moderately correlated with objective income (Grable et al., 2013). Therefore, this perceptual assessment is important in that it can capture a variety of circumstances, having an income that does or does not meet one’s desires, regardless of how objectively “high” or “low” the income may be. PIA has been related to both personal well-being and organizational attitudes in prior research (Cheung, 2014; Sears, 2008). Viewing PIA as a critical and valued resource, we predicted higher PIA would be associated with better mental and physical health, which we operationalize as self-reports of depression symptoms and physical health complaints.

- *Hypothesis 1: *PIA is negatively related to depression and physical health symptoms.

## PIA and Recovery

Recovery has been studied, often in the context of COR theory, as a mechanism for replenishing lost resources which protect workers from strain (Sonnentag et al., 2017). Recovery has been examined in a variety of forms (e.g., breaks, vacations; Fritz et al., 2013; Sonnentag et al., 2017); our focus was general recovery experiences outside of regular work time. Early research into recovery identified leisure activities, such as social activities, physical activities, and low effort activities (e.g., watching television, reading) that were associated with well-being benefits (Sonnentag, 2001). However, individuals can engage in such activities without really experiencing recovery (Fritz et al., 2013).

In a framework that has since become widely adopted, Sonnentag and Fritz (2007) developed a measure of four specific qualities of recovery experiences that can occur during various leisure activities. They found that recovery experiences involving psychological detachment (e.g., not thinking about work), mastery (e.g., learning a new skill), control (i.e., over one’s schedule), and relaxation were each associated with better health and well-being. A good deal of research has documented health-related benefits of each recovery experience (Headrick et al., ﻿^2023^). Psychological detachment and relaxation activities in particular have demonstrated an important and consistent role in buffering the effects of job demands on well-being (Sonnentag et al., 2010, 2017). Consistent with propositions of COR theory, the inability to replenish resources can result in cyclical relationships, affecting both recovery experiences in subsequent days and fatigue (von Thiele Schwarz, 2011).

COR theory suggests that individuals lacking resources or experiencing strain would take steps to replenish resources. Thus, it makes sense that one experiencing strain would seek out recovery experiences as valued resources. However, individuals may be faced with situations where efforts to seek out one resource are not compatible with attempts to restore another. Applied to the present study, taking time for recovery is a resource-accumulating behavior that could protect workers’ health and well-being, but the perceived insufficiency of income and need for monetary resources is likely to feel more salient and urgent, given the need for money for survival in modern society. This idea is captured in theories of resource scarcity. Specifically, how individuals allocate their time may be affected by the scarcity of financial resources (Grossman & Mendoza, 2003) and a focus on the scarcity of financial resources can result in attention being devoted to money matters at the neglect of other areas (Mani et al., 2020). In otherwise neutral circumstances, Shah and colleagues (2018) found that low-income individuals had more money-related thoughts than higher-income individuals. This is further paralleled by motivational theories, which highlight that needs that are most intense are likely to be those that individuals work to satisfy first (e.g., Steel et al., 2006). Related, Hobfoll (1989) acknowledges that the value placed on a resource may affect one’s efforts to obtain/restore it. Taken together, these past findings and theory suggest that individuals with an adequate income may feel free to pursue additional resource replenishment through recovery experiences (i.e., gain spirals), which can benefit their health and well-being. Alternatively, those with an inadequate income may feel pressure to focus on restoring financial resources (e.g., seeking more work hours) rather than seeking enjoyable leisure, which may feel secondary. This is especially true for many recovery activities that require money (e.g., gym memberships; supplies for hobbies) and more broadly true of all recovery that involves a time investment, which may feel costly if it is at the expense of time spent on activities with income-earning potential. Taken together, we expected that individuals with a more sufficient income will be more likely to report recovery experiences, feeling able to invest in additional resource acquisition.

- *Hypothesis 2*: PIA is positively related to recovery experiences.

Following from the findings on the influence of scarcity of resources on cognition, another barrier to recovery and receiving actual benefits from recovery might be one’s mindset toward taking an opportunity for rest or other non-work related, desirable activities. Individual differences, such as personal mindfulness, general activity preferences, and affective dispositions, have been found to explain some of the connections between recovery and well-being in past research (Hunter & Wu, 2016; Marzuq & Drach-Zahavy, 2012; Ragsdale et al., 2016). Further, one’s financial situation has been found to relate to thoughts about everyday situations, such as spending time with friends or going out to dinner (Shah et al., 2018). Thus, thoughts about money are likely to relate to the perceived benefit or value of recovery, and alternatively, guilt that could coincide with attempts to recover. Relaxation remorse, feeling guilty for engaging in relaxing activities rather than productive activities related to work, is related to more psychological health symptoms, more perceived stress, and fewer recovery experiences (Black & Britt, 2023). Such remorse could theoretically be experienced in response to personal or situational factors that change the value placed on recovery. As previously discussed in the context of COR theory, individuals must at times make tradeoffs in what resources they pursue. Pursuing financial resources may feel more valuable than pursuing psychological resources through recovery experiences when income is perceived as inadequate. Further, engaging in recovery activities in a context where financial resources feel scarce and money is a salient factor to an individual’s thoughts and behaviors (e.g., Mani et al., 2020; Shah et al., 2018), not allocating time and energy toward filling a financial need may even bring distress. Therefore, we expected that perceiving a more sufficient income would not only be related to more recovery experiences, but also a more positive attitude toward taking time for relaxation.

- *Hyphothesis 3*: PIA is negatively related to relaxation remorse.

## Indirect Relationships between PIA and Health via Relaxation Remorse and Recovery

Together, these connections between PIA and recovery may partially explain why one’s financial situation relates to physical and mental health. We acknowledge that there are several existing mechanisms that can explain why an insufficient income could negatively affect health (e.g., access to practical health and educational resources, Williams, 1990; health beahviors, Prentice et al., 2017; perceived control and future outlook; Creed & Klisch, 2005; Price et al., 2002; Richter & Näswall, 2018). However, no studies have sought to connect PIA to one’s mindset and practices of recovery. Studies have suggested that having to be available for work for extended periods of time can harm well-being through a lack of recovery, particularly because of low control over available recovery time (Dettmers et al., 2016). Paired with scarcity perspectives and the salience of resource needs previously discussed, it is likely that PIA could also impact health through the engagement in recovery activities and/or one’s mindset toward such activities.

We specifically explored whether there was an indirect relationship between PIA and health outcomes via the recovery-related attitude of relaxation remorse and recovery experiences. For example, an insufficient income could lead individuals to neglect recovery either out of necessity (i.e., because they are actually working additional hours to obtain financial resources) or because of guilt (i.e., feeling that taking a break is undeserved when income-related resources are lacking, and that resource need is salient). We tested whether relaxation remorse and the different types of recovery experiences could explain some of the variability in the relationship between PIA and health, within a parallel indirect effects model.^1^ We note that in these analyses, we separated the four types of recovery experiences to explore whether the proposed relationships would emerge with all recovery experiences, or if nuances would be present in which some, but not all, types of recovery experiences help explain the PIA – health relationships. In sum, we proposed the following hypotheses regarding indirect effects.

- *Hypothesis 4a:* There is an indirect relationship between PIA and health symptoms via relaxation remorse (i.e., those with sufficient income will feel less remorse for relaxing, which results in fewer health symptoms).
- *Hypothesis 4b: *There is an indirect relationship between PIA and health symptoms via recovery experiences (relaxation, detachment, mastery, control; i.e., those with sufficient income will have more recovery experiences, which results in fewer health symptoms).

## Method

### Participants and Procedure

These data were gathered as part of a larger scale validation study (Black & Britt, 2023)*.* As described earlier, we included PIA with the secondary goal of exploring connections between one’s financial situation and recovery, secondary to the primary study purpose of scale validation. A full data transparency statement is provided in Appendix 1.

The sample consisted of employed adults who were recruited using Amazon’s Mechanical Turk (MTurk). A request to complete a survey about perceptions around workplace stress was posted to MTurk. After providing informed consent and agreeing that study qualifications were met (i.e., being a U.S. Citizen, 18 years of age or older, and employed in a job outside of MTurk for at least 30 h per week), the participants proceeded to the survey.

A total of 1,126 employees completed the survey. A small portion of participants (N = 49 or 4%) either completed the survey in an unreasonable amount of time or failed at least one of four attention check items that were included throughout the survey to improve data quality (Cheung et al., 2016). An example attention check item was, “Please select "agree" for this item”. Thus, 1,077 participants completed the entire survey and received $2.00 in compensation.

All 1,077 participants were contacted two months following the first survey using communications on MTurk and the MTurk worker IDs provided on the Time 1 survey. They were invited to complete a second survey, again assessing stress perceptions, health, and job-related variables. While a two-month time frame may not show the nuanced impacts of daily recovery, we felt this could provide a sufficient time lag to see impacts of PIA and recovery-related variables on health outcomes. Further, separating measurement occasions could reduce some concerns with common method variance compared to all data being collected at once (Podsakoff et al., 2003). In total, 789 (73%) of those participants who were invited did complete the Time 2 survey for an additional $2.00. After removing participants that failed an attention check and those that could not be matched based on the MTurk IDs provided in the second survey (*n* = 37) there were 752 participants that could be matched. Additional data cleaning procedures were conducted after data collection was completed to enhance data quality. Specifically, duplicate IP addresses or/or MTurk IDs were removed (*n* = 80) and cases with zero intra-individual response variability within a scale mid-way through the surveys that included reverse worded items were also removed (*n* = 19; Dunn et al., 2016). This resulted in a final matched sample of 683.

In the matched sample participants ranged in age from 18 to 71, with a mean of 37 years (*SD* = 10.55). Participants’ reported sex was roughly equivalent between males (56%) and females (44%). The sample was predominantly White/Caucasian (84%); the remaining participants were Black/African American (8%), Asian (5%), other (2%), and American Indian or Alaska Native (less than 1%). Most participants had received either a bachelor’s degree (39%) or some college education (25%), and the remaining had a post-graduate degree (14%), Associate’s or two-year technical degree (11%), GED (11%), or some high school education (< 1%). Participants worked in a variety of occupational fields, with common fields including sales-related, computer and mathematical, and education, training, and library.

To examine potential attrition bias, chi-square tests and independent samples t-tests were conducted to compare demographics and primary predictor variables between participants in the matched sample and those who completed the Time 1 survey but did not respond to the Time 2 survey. There were no differences in reported gender, race, or occupational groups. Results did indicate a small, but significant difference in education, *χ*^2^ (5, *N* = 998) = 11.74, *p* = 0.04, Cramer’s V = 0.11, with the matched sample having higher representation of post-graduate degrees. On average, participants in the matched sample (*M* = 36.65) were older than the Time 1 only sample (*M* = 32.79; *t* (987) = 5.18, *p* < 0.05). There were no significant differences in PIA, but small differences in relaxation remorse between the matched (*M* = 4.08) and Time 1 only sample (*M* = 4.32; *t* (988) = 2.05, *p* = 0.04), but the effect was small (*d* = 0.14). Given the non-significant or small differences, we did not see evidence for substantial attrition bias.

## Measures

### Perceived Income Adequacy (PIA)

The items used to measure PIA (Sears, 2008) at Time 1 assessed the degree to which an individual felt their income level was sufficient. The scale included 10 items scored on a five-point Likert scale (1 = *strongly disagree* to 5 = *strongly agree)*. This scale included statements such as “I am currently able to meet my financial goals” and “I can afford the food I need to survive”. The measure demonstrated high internal consistency (Cronbach’s *α* = 0.92).

### Relaxation Remorse

Relaxation remorse was measured at Time 1 and Time 2 using items developed by Black and Britt (2023). The six-item scale evaluated the degree to which an individual generally felt guilty when disengaging from work-related activities during non-work time. It was scored on a seven-point Likert scale (1 = *strongly disagree* to 7 = *strongly agree)*. Individual items included statements such as, “Relaxing makes me feel guilty because there is always something else I could be doing for work”. This scale had high internal consistency (Cronbach’s *α* = 0.96).

### Recovery Experiences

Recovery experiences were measured at Time 1 and Time 2 using the Recovery Experiences Questionnaire (Sonnentag &Fritz, 2007), which addressed an individual’s experienced relaxation, psychological detachment, control, and mastery during time outside of work. Participants were asked, in reference to their free evenings or time away from work, the extent to which they agreed with 16 items. Each of the four subscales contained four items, all of which were scored on a five-point Likert scale (1 = *strongly disagree* to 5 = *strongly agree*). The *relaxation* subscale included statements such as, “I take time for leisure” (Cronbach’s *α* = 0.90). The *psychological detachment* subscale included statements such as, “I forget about work” (Cronbach’s *α* = 0.82). The *control* subscale included statements such as, “I determine for myself how I will spend my time” (Cronbach’s *α* = 0.78). The *mastery* subscale included statements such as, “I do things that challenge me” (Cronbach’s *α* = 0.86).

### Health Symptoms

#### Physical health symptoms

Symptoms of physical health problems were assessed at Time 2 using Spector and Jex’s (1998) Physical Symptoms Inventory (PSI). The PSI includes a list of 18 health complaints, such as “an upset stomach or nausea” and “eye strain.” Participants were asked to indicate first, whether they had experienced each symptom in the past six weeks (yes or no), and second, whether they had seen a doctor for the symptom in the past six weeks (yes or no). Responses to both the experience of symptoms items and whether or not the individual saw a doctor were summed together for an overall frequency/severity measure of physical health symptoms. Higher scores represented more symptoms and/or doctor visits. Internal consistency measures were not computed because the construct is formative in nature.

#### Depression

Depression symptoms were measured at Time 2 using the Patient Health Questionnaire-9 (Spitzer et al., 1999). This scale evaluated the degree to which an individual experienced depressive-type symptoms during the two weeks prior to the self-report. It consisted of nine items scored on a four-point Likert scale, where 0 indicated *not at all* and 3 indicated *nearly every day*. The PHQ-9 included statements such as “Little interest or pleasure in doing things” and “Feeling tired or having little energy” (Cronbach’s *α* = 0.92).

### Demographic and Control Variables

Several demographic variables were collected and considered as control variables, given research that does show some group differences in mental and physical health or health-reporting tendencies based on age, gender, and race (e.g., Adler & Rehkoph, 2008; Gijsbers van Wijka et al., 1999; Pinquart, 2001; Tesch- Römer et al., 2007). Gender and race were dichotomized as male/female (no individuals identified as other) and white/non-white. We also controlled for work hours to account for work demands differences that could impact health. Lastly, we controlled for general positive and negative affect, as general affect can be strongly correlated with health, particularly mental health (Cohen & Pressman, 2006; Watson et al., 1988). Positive and negative affect were measured with the positive and negative affect schedule (PANAS; Watson et al., 1988). Items asked in general, how often individuals felt 10 positive emotions (e.g., interested, excited) and 10 negative emotions (e.g., irritable, distressed) with ratings on a 1 (very slightly or not at all) to 5 (extremely) scale. Both scales demonstrated acceptable reliability (*α* = 0.92 for positive and 0.93 for negative).

### Data analytic plan

Analyses were conducted in R Studio using the lavaan package (Rosseel, 2012). First, a measurement model was tested with all reflective measures. The maximum likelihood imputation option provided in lavaan was applied to handle missing data, to use full sample information (though we note we had very few missing responses and results were similar when computed with listwise deletion). Robust maximum likelihood estimation was used to account for non-normality in the data (Hu & Bentler, 1992). As general guidelines, but not strict cutoffs, for acceptable fit, we used recommendations by Hu and Bentler (1999): CFI greater than 0.95, RMSEA lower than 0.06, and the SRMR lower than 0.08.

To test our study hypotheses, we used structural equation modelling. We specified models to test the direct and indirect relationships among PIA and health outcomes via relaxation remorse and recovery experiences. We controlled for the effects of age, gender, work hours, and positive and negative affect on the health outcomes; race was omitted as a covariate, as it did not significantly relate to the health outcomes. We tested two structural models: one which examined the relationships between PIA and health outcomes (Hypothesis 1) and one which then incorporated relationships with relaxation remorse and the four recovery experiences as parallel mediators, testing Hypotheses 2, 3, 4a, and 4b. The primary predictor, PIA, was measured at Time 1 and the distal outcomes (depression, physical health symptoms) were measured at Time 2. For testing indirect effects, we first tested a model with relaxation remorse measured at Time 1 and recovery experiences measured at Time 2. We also tested alternative indirect models with all mediators assessed at Time 1, all mediators assessed at Time 2, and mediators assessed at Time 2, controlling for Time 1 reports.

## Results

In establishing a measurement model for the primary analyses, a seven-factor model was tested with PIA (Time 1), relaxation remorse (Time 1), the four recovery experiences (Time 2), and depression (Time 2). This model exhibited acceptable fit (χ^2^ (758) = 1952.16, CFI = 0.92, RMSEA = 0.055, 90% CI 0.052, 0.058, SRMR = 0.047). Although the CFI was somewhat lower than ideal, we retained this model without adding additional alterations. All standardized factor loadings exceeded 0.60, except for one depression item and one recovery (control) item. Since these are previously validated and widely used scales, we retained these items to be consistent with the literature. A more parsimonious model, combining the four recovery experiences into one factor, was also tested, but demonstrated worse fit (χ^2^ (773) = 3475.357, CFI = 0.81, RMSEA = 0.082, 90% CI 0.079, 0.085, SRMR = 0.078) compared to the seven-factor model.

Potential covariates and the composite score for physical health were added to compute basic correlations among all study variables. These correlations are provided in Table 1. Providing initial support for Hypothesis 1, PIA was negatively correlated with physical health symptoms (*r* = -0.28, *p* < 0.05) and depression symptoms (*r* = -0.38, *p* < 0.05). Correlations between PIA and all four recovery experiences were small, but significant (*r* range 0.11 to 0.19, *p* < 0.05), providing initial support for Hypothesis 2. PIA also related to less relaxation remorse (*r* = -0.14, *p* < 0.05), providing initial support for Hypothesis 3. As preliminary support for the proposed indirect paths, both relaxation remorse and the four recovery experiences were significantly correlated with health symptoms. Relaxation remorse was also correlated with fewer recovery experiences, apart from a non-significant correlation with mastery.

**Table 1:** Descriptive Statistics and Correlations among primary study variables

Variable	Mean	SD	1	2	3	4	5	6	7	8	9	10	11	12	13	14
1. Age	36.65	10.63	–													
2. Gender	–	–	.13*	–												
3. Race	–	–	-.11*	.11*	–											
4. Work hours	42.06	6.52	.03	-.18*	.02	–										
5. PA	3.25	0.87	.11*	-.05	.05	.16*	(.92)									
6. NA	1.50	0.69	-.18*	.06	.08	.00	-.15*	(.93)								
7. PIA	3.64	0.83	.05	.16*	-.04	.08	.26*	-.34*	(.92)							
8. Relaxation Remorse	4.08	1.73	-.03	.19*	-.01	.13*	.06	.25*	-.14*	(.96)						
9 Relaxation Activities (T2)	3.75	0.83	.02	-.08*	.03	-.13*	.05	-.20	.18*	-.48*	(.90)					
10. Detachment (T2)	3.17	0.92	.01	-.09*	.03	-.19*	-.05	-.09	.11*	-.48*	.70*	(.82)				
11. Control (T2)	3.98	0.66	.04	.02	.06	-.09	.16*	-.20*	.19*	-.26*	.59*	.48*	(.78)			
12. Mastery (T2)	3.77	0.75	-.02	-.06	.04	.12*	.35*	-.15*	.11*	.06	.03	-.11*	.24*	(.86)		
13. Physical Symptoms (T2)	4.74	4.04	-.03	.23*	-.03	-.04	-.16*	.36*	-.28*	.26*	-.19*	-.19*	-.16*	-.10*	–	
14. Depression (T2)	0.56	0.64	-.20*	.11*	.02	.00	-.26*	.65*	-.38*	.30*	-.27*	-.18*	-.29*	-.12*	.51*	(.92)

Notes: * p < *.05. N* = *683.* Variables were measured at time 1 unless noted otherwise with (T2). Reflective scales are modeled as latent variables. Gender was coded as 1 = male, 2 = female. Race coded as 1 = white, 2 = non-white. PA = positive affect. NA = negative affect. PIA = perceived income adequacy. Values in parentheses along the diagonal indicate estimates of Cronbach's Alpha

Next, we generated SEM models to examine the direct and indirect effects. First, we focused on the relationship between PIA and health outcomes, and found further support for Hypothesis 1 in a more stringent model. Even after including demographic covariates and positive and negative affect, PIA was significantly related to fewer physical health (*b* = -0.56, *se* = 0.18, β = -0.13, *p* = 0.002) and depression symptoms (*b* = -0.11, *se* = 0.03, β = -0.14, p < 0.001).

**Fig. 1:**
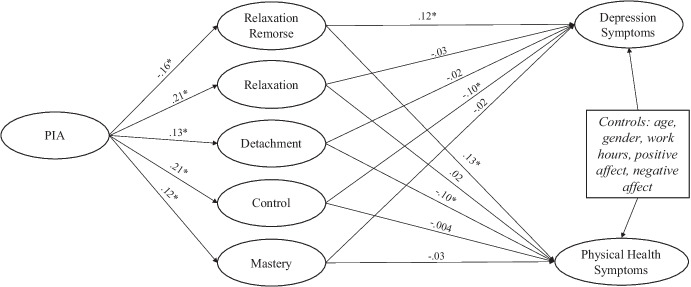
*Parallel indirect effects of PIA on health outcomes *via* relaxation remorse and recovery activities. Note.* Figure displays standardized regression coefficients (*β*). PIA and Relaxation remorse measured at Time 1; recovery experiences and health symptoms measured at Time 2. While not pictured, the direct effect of PIA remained significant *β* = -.11, p = .007 for depression; *β* = -.11, p = .01 for physical health

Next, we added in the parallel indirect effects via relaxation remorse and the four recovery experiences (summarized in Fig. 1). In support of Hypothesis 2, PIA was related to more recovery experiences: relaxation (*b* = 0.19, *se* = 0.04, β = 0.21, p < 0.001), detachment (*b* = 0.14, *se* = 0.05, β = 0.13, p = 0.008), control (*b* = 0.14, *se* = 0.04, β = 0.21, p < 0.001), and mastery (*b* = 0.08, *se* = 0.04, β = 0.12, *p* = 0.02) during non-work time. Supporting Hypothesis 3, PIA was related to less relaxation remorse (*b* = -0.32, *se* = 0.09, β = -0.16, p < 0.001). Thus, perceiving an adequate income does seem to correlate with greater participation in recovery activities and less guilt for time spent in relaxation. Connecting relaxation remorse and recovery experiences to the health outcomes, only relaxation remorse (*b* = 0.05, *se* = 0.02, β = 0.12, p = 0.001) and control (*b* = -0.10, *se* = 0.05, β = -0.10, p = 0.048) were significantly related to depression. Only relaxation remorse (*b* = 0.27, *se* = 0.10, β = 0.13, *p* = 0.004) and detachment (*b* = 0.41, *se* = 0.20, β = -0.10, p = 0.04) were related to physical health symptoms.

When examining the significance of the indirect effects (summarized in Table 2), there was a significant indirect effect of PIA on depression and physical health symptoms via relaxation remorse. Alternatively, the indirect effects via recovery experiences were largely non-significant. Only the indirect effect of PIA on depression via control approached significance (*p* = 0.06). In alternative models, the indirect effect through relaxation remorse was significant when using Time 1 or Time 2 measurements. Further, the significance of the indirect effects was marginal (*p* = 0.07 for depression; *p* = 0.05 for physical health) in a more stringent analysis where relaxation remorse at Time 2 was included as a mediator while also controlling for relaxation remorse and recovery experiences at Time 1. This provides some evidence that changes in relaxation remorse could be one factor connecting PIA and health; however, given the marginal significance, we caution against drawing firm conclusions from these data. The only indirect relationship that approached significance when using Time 1 or Time 2 mediators for recovery experiences was the previously mentioned relationship of PIA via control affecting depression. In sum, we found support for Hypothesis 4a but not 4b.

**Table 2:** Indirect effect models examining the effects of PIA (Time 1) on health (Time 2) via relaxation remorse and recovery experiences

*Relaxation remorse measured at Time 1 and recovery experiences measured at Time 2*				
	*Indirect Effect*	*SE*	*z-value*	*p(* >*|z|)*
**PIA > RR > Depression**	**-0.015**	**0.006**	**-2.286**	**0.022**
**PIA > RR > Physical**	**-0.086**	**0.039**	**-2.198**	**0.028**
PIA > Relaxation > Depression	-0.004	0.008	-0.522	0.602
PIA > Relaxation > Physical	0.014	0.052	0.268	0.789
PIA > Detachment > Depression	-0.002	0.004	-0.392	0.695
PIA > Detachment > Physical	-0.056	0.034	-1.623	0.105
**PIA > Control > Depression**	**-0.015**	**0.008**	**-1.919**	**0.055**
PIA > Control > Physical	-0.004	0.052	-0.077	0.939
PIA > Mastery > Depression	0.002	0.003	0.518	0.604
PIA > Mastery > Physical	-0.015	0.021	-0.742	0.458
*All mediators measured at Time 1*				
	*Indirect Effect*	*SE*	*z-value*	*p(* >*|z|)*
**PIA > RR > Depression**	**-0.019**	**0.008**	**-2.397**	**0.017**
**PIA > RR > Physical**	**-0.089**	**0.043**	**-2.079**	**0.038**
PIA > Relaxation > Depression	-0.001	0.006	-0.097	0.923
PIA > Relaxation > Physical	0.007	0.037	0.186	0.852
PIA > Detachment > Depression	0.001	0.002	0.391	0.696
PIA > Detachment > Physical	-0.016	0.021	-0.773	0.44
PIA > Control > Depression	-0.002	0.008	-0.242	0.809
PIA > Control > Physical	0.007	0.053	0.134	0.894
PIA > Mastery > Depression	0.002	0.003	0.597	0.551
PIA > Mastery > Physical	0.005	0.015	0.319	0.75
*All mediators measured at Time 2*				
	*Indirect Effect*	*SE*	*z-value*	*p(* >*|z|)*
**PIA > RR > Depression**	**-0.021**	**0.009**	**-2.328**	**0.02**
**PIA > RR > Physical**	**-0.141**	**0.054**	**-2.62**	**0.009**
PIA > Relaxation > Depression	-0.003	0.009	-0.348	0.728
PIA > Relaxation > Physical	0.024	0.053	0.462	0.644
PIA > Detachment > Depression	-0.001	0.004	-0.311	0.756
PIA > Detachment > Physical	-0.052	0.033	-1.563	0.118
**PIA > Control > Depression**	**-0.015**	**0.008**	**-1.875**	**0.061**
PIA > Control > Physical	-0.004	0.052	-0.077	0.939
PIA > Mastery > Depression	0.002	0.003	0.439	0.661
PIA > Mastery > Physical	-0.017	0.021	-0.828	0.408
*Time 2 Relaxation remorse, controlling for Time 1 relaxation remorse and recovery experiences*				
	*Indirect Effect*	*SE*	*z-value*	*p(* >*|z|)*
**PIA > RR > Depression**	**-0.017**	**0.01**	**-1.794**	**0.073**

*Notes:* All models control for the effects of age, gender, hours worked, negative affect, and positive affect on health outcomes. Significant (p < .05) and marginally significant (p < .10) indirect effects are indicated in bold. PIA = perceived income adequacy. RR = relaxation remorse

## Discussion

The purpose of the present study was to explore connections between perceptions of income adequacy, feelings of guilt caused by disengaging from work-related demands during off-work time, participation in recovery experiences, and health outcomes. In alignment with existing literature which finds that one’s financial situation can impact worker health and well-being (e.g., Cheng & Chan, 2008; Prentice et al., 2017; Price et al., 2002; Richter & Näswall, 2018), the current study provided evidence for a negative relationship between PIA and both physical and mental health symptoms (Hypothesis 1). These findings affirm the notion that resources, specifically adequate perceived monetary resources, can be protective for one’s health. Perceptions of one’s ability to afford basic wants and needs in particular, is an important perceptual resource to continue to consider as a contributor to personal well-being.

There was evidence for positive relationships between PIA and recovery experiences (Hypothesis 2). On the other hand, there was a negative relationship between PIA and feelings of relaxation remorse (Hypothesis 3). Summarizing this, those with a more adequate income seem to feel more comfortable relaxing and tend to report more quality elements of recovery. These results align with key tenets of COR theory (Hobfoll, 1989, 2001), which purports that having resources makes it easier to gain resources. Thus, when income is perceived as adequate, individuals are better situated to engage in recovery, acquiring more psychological resources. When confronted with stress, such as that which is likely to arise when one perceives their income as insufficient or scarce, individuals may place less priority on relaxation and recovery, seeing this opportunity for resources as less urgent than the need for monetary resources (e.g., Shah et al., 2018). Although income and energy gained from recovery are both considered key resources, it is likely that individuals perceive monetary resources as more important. The importance of monetary resources is emphasized in scarcity perspectives of financial stress (e.g., Mani et al., 2020). This line of thinking could lead to acceptance of burnout and prioritization of work-related activities over recovery. This trend may be especially prominent when individuals feel threatened by potential cycles of resource loss (i.e., lack of money could then lead to an inability to provide for oneself and/or one’s family, loss of stable living environment, loss of personal esteem, etc.).

Considering the indirect effects, there was support for relaxation remorse explaining a portion of the relationships between PIA and health outcomes (Hypothesis 4a), but very little support for recovery experiences explaining those relationships (Hypothesis 4b). Only control approached significance. Variations of the mediation model provided some support that the indirect effects via relaxation remorse, but not recovery experiences, are likely not largely impacted by common method variance attributed to using the same measurement occasion since these effects held whether Time 1 or Time 2 measurements were used. Thus, it may be that PIA is more influential on health via on one’s mindset toward recovery than actual recovery behaviors, given recovery experiences could also be affected by a wide variety of factors (e.g., family or community obligations, accessibility of hobbies, etc.). Still, given the rich literature supporting the connection between recovery experiences and well-being, it is surprising to see the relationships between recovery experiences and health outcomes were largely non-significant when also accounting for relaxation remorse.

Further considering the importance of relaxation remorse, one’s mindset toward recovery may be one of the critical areas that one’s perceived financial situation impacts. An adequate income could allow an individual the freedom to pursue this additional resource of recovery without guilt or remorse, resulting in benefits for their well-being. Alternatively, an inadequate income could cause individuals to devalue recovery and consider relaxation to be a waste of time or simply a luxury when monetary resource needs are not met. More broadly, it is important to further explore this perception of relaxation remorse which could lead individuals to neglect participation in efforts to try to restore a sense of balance. Such a scenario was supported by the generally significant correlations between relaxation remorse and relaxation, detachment, and control (but not mastery).

The lack of significant unique and mediating relationships with recovery experiences other than control was still surprising, given the fact that psychological detachment and relaxation activities have shown more consistent relationships with strain and well-being in past research (Sonnentag et al., 2017). The one marginal indirect effect via control suggests that PIA may be associated with taking an empowered approach to recovery (i.e., feeling personal control) which can yield valuable psychological benefits. In particular, income adequacy may “free up” an individual to feel a greater sense of control in non-work time, facilitating the health benefits of recovery time. Alternatively, those with an inadequate income may feel more constraints in truly enjoying their non-work time, particularly if it is impacted by taking on additional work tasks or additional jobs to try to earn more money. A narrowed focus on income or income-earning activites is supported by scarcity perspectives, where individual’s can develop a tunnel-vision focus on their lack of financial resources (Grossman & Mendoza, 2003; Mani et al. 2020). These findings also complement the work of Dettmers et al. (2016), who found that having extended on-call hours affected worker’s ability to have recovery experiences through diminished feelings of control.

## Theoretical and Practical Implications

The present study makes an important contribution to the literature by bridging research on financial resources and recovery. While both financial variables and recovery have been shown to impact health and well-being, no studies to our knowledge have sought to integrate these areas. We believe this is critical, as the literature often promotes recovery as an important antidote to stress. Our study is one of the first to consider whether one’s perceived financial situation could act as a gateway to obtaining those benefits of recovery experiences without guilt.

While there are numerous factors that can connect income with health and well-being, our study adds a novel area that shows that income adequacy may alter one’s mindset toward taking time for relaxation, ultimately promoting better health. This study also provides support for the tenant of COR theory which proposes that having resources makes it easier to acquire additional resources. In the case of our study, PIA was related to more recovery experiences and less remorse around relaxation. Thus, this perceptual resource around finances can facilitate the accumulation of additional psychological and energetic resources.

Our study also highlights the importance of an adequate income to one’s health and well-being. The direct effects of PIA on health were persistent, even in models accounting for covariates and the recovery-related variables. Thus, a primary implication of our study results is the need for organizations to pay employees an income that is adequate to meet their needs. Income adequacy is perceptual, so other tools, such as personal finance education could also be valuable for individuals who may simply need assistance in utilizing finances more effectively to meet their wants and needs.

Although other variables, like those concerning access to resources, may explain more variance in health outcomes, the existence of the indirect path through relaxation remorse also suggest avenues for intervention for workers experiencing income-related stress. Recovery experiences can be improved through intervention programs (e.g., Ebert et al., 2015; Hahn et al., 2011; Siu et al., 2014), but such interventions may be ineffective if an individual views relaxation as inaccessible to them. These programs may be most effective if basic resource needs are met (i.e., sufficient income) and they are able to combat perceptions of relaxation remorse that could interfere with the desire to use these skills. Workers may also need more detailed interventions helping them understand the costs and benefits of focusing on replenishing income verses psychological resources and even how replenishing psychological resources could protect their health, enabling them to be better situated to acquire more income, given the relationships between mental and physical health and job performance outcomes (Ford et al., 2011). Some workers who still must work long hours or multiple jobs may need more targeted intervention strategies for brief, but effective recovery methods that would evoke minimal guilt for taking time to recover.

## Limitations and Future Directions

The current study is not without limitations, which should be considered in interpreting and applying our findings. Because participants provided self-report data, common-method variance may present an issue. However, the design of this study served as one method to buffer the influence of common-method variance through the use of Time 1 and Time 2 surveys, separating the measurement of the independent and dependent variables by two months (Podsakoff et al., 2003). Still, separate measurements are not a perfect solution (Brawley Newlin, 2023). For future research, we recommend that researchers also explore the use of more objective measures, such as income-to-expense ratios, in further studies regarding PIA. Physiological data such as sleep activity, heart rate, blood pressure, or cortisol levels could also be strong indicators of health and even evidence of relaxation and recovery.

Additionally, some of our effects, though significant, were relatively small in magnitude. Given we did have a sample size that should have been adequate to detect the hypothesized effects, these small but significant effects merit further investigation and replication. In particular, replication with varied time frames is important. Since recovery is a process that may be affected by day-level events, within-subjects designs with repeated measurements of the variables of interest could be influential. Specifically, studies could look at whether everyday thoughts about the sufficiency of income affect same day recovery practices. Such studies could also connect feelings of relaxation remorse to specific instances of recovery, as opposed to our study that relied on more general reports of these variables verses more specific situational measures. On the other hand, studies with an extended time frame may be more likely to see effects that may take time to accumulate, such as the effects of income or insufficient recovery on health outcomes.

Lastly, our use of only two time points does not provide an optimal test for mediated effects. These data were part of a larger scale validation study, where the primary purpose of the study was not designed to optimally test mediation effects. Still, the collected variables offered an opportunity to explore the novel relationships, albeit with an imperfect design. Ideally our study could be replicated with all measures at different time points and a fully longitudinal design with all variables measured at each time point. Though there is a logical rationale that perceptions of income would proceed decisions to engage in recovery/mindsets toward recovery compared to the reverse (i.e., recovery prompting one’s appraisal of their income adequacy), causal effects cannot be determined with the design used in our study.

Despite its limitations, the current study connects important areas of research, revealing possible connections between one’s perceived financial situation, recovery, and health. More detailed investigations with additional metrics of one’s financial situation and comparing participation in active versus passive recovery strategies for individuals who feel a strong need to protect against monetary resource loss would provide a solid foundation for future studies in this area. There may also be an underlying impact of personality on effectiveness of recovery strategies when under some degree of financial strain, varying from individual to individual. Examination of this possibility and methods by which to optimize recovery in the presence of financial inadequacy on an individual level could provide an interesting area of related study.

## Conclusion

The purpose of the present study was to investigate possible associations between an individual’s perceived financial situation and their personal health, through mechanisms of both relaxation remorse and recovery experiences. This study allowed for evaluation of potential psychosocial factors that may explain the relationship between one’s perceived financial situation and health outcomes. We found that the adequacy of one’s income does relate to one’s mindset toward relaxation and, to a lesser degree, recovery experiences, further affecting several health outcomes. Our findings highlight the relationships that income-related factors could have with personal recovery, as well as the potential need for unique recovery strategies to help workers truly recover and not feel remorse for taking time for a break.

## Data Availability

The data that support the findings of this study are available from the corresponding author upon reasonable request.

## Appendix 1

### Data Transparency Statement

For transparency, the authors wish to disclose that portions of the data were used in a published paper (Black & Britt, 2023). This manuscript was a measure development paper, focused on developing and providing validity evidence for a measure of stress as a badge of honor, which included relaxation remorse as one dimension. Qualitative findings from this initial study indicated that participants cited financial reasons for feeling guilt for taking time for recovery activities. Thus, in later stages of the measure development project, we included measures of PIA to explore a secondary research question about the relationships between income and recovery. We believe that this does not present concerns for redundancy in the use of the data because:

- the initial and primary purpose of the published paper was to conduct a construct validity study; analyses were primarily correlations without more involved model testing.
- the current manuscript focuses on PIA as primary predictor of health and well-being, utilizing one of the subscales (i.e., relaxation remorse) validated in the first study as a mediating variable, rather than focal predictor.
- The only redundant relationships incorporated into both papers is the relationship between relaxation remorse (T1) and health (T2), which were reported in the earlier paper. Since the focus of this paper was on PIA related to health and PIA related to relaxation remorse/recovery, we still felt this was an interesting and novel contribution.

(Table 3).

**Table 3:** The table below summarizes the uses of variables in the two papers

	Published paper	Current Paper
Perceived Income Adequacy		X
Stress as achievement	X	
Relaxation remorse	X	X
Stress-related impression management	X	
Stress-related social comparisons	X	
Positive affect	X	
Negative affect	X	
Social desirability	X	
Workaholism	X	
Perfectionism	X	
General social comparison	X	
Recovery Experiences	X (only T1 relaxation & detachment)	X (all 4 subscales at T2 + T1 incorporated for alternative models)
Performance	X	
Anxiety	X	
Depression	X	X
Physical Health	X	X
Work-family conflict	X	
Relationship quality	X	
Perceived Stress	X	

## Footnotes

1 Serial mediation was also explored in three-path models; however, these were largely unsupported and not optimally tested with our study design. Thus, these findings are omitted for simplicity. Those results can be requested from the first author.
